# Prevalence of Metastatic Lateral Lymph Nodes in Asian Patients with Lateral Lymph Node Dissection for Rectal Cancer: A Meta-analysis

**DOI:** 10.1007/s00268-021-05956-1

**Published:** 2021-02-04

**Authors:** Niki Christou, Jeremy Meyer, Christophe Combescure, Alexandre Balaphas, Joan Robert-Yap, Nicolas C. Buchs, Frédéric Ris

**Affiliations:** 1 Digestive Surgery Department, University Hospital of Limoges, 2 Avenue Martin Luther King, 87042 Limoges, France; 2 Division of Digestive Surgery, University Hospitals of Geneva, Rue Gabrielle-Perret-Gentil, 41211 Geneva 14, Switzerland; 3 Unit of Surgical Research, University of Geneva, Rue Michel-Servet 1, 1206 Geneva, Switzerland; 4 Division of Clinical Epidemiology, University Hospitals of Geneva, Rue Gabrielle-Perret-Gentil 4, 1211 Geneva 14, Switzerland

## Abstract

**Importance:**

Rectal cancers occupy the eighth position worldwide for new cases and deaths for both men and women. These cancers have a high tendency to form metastases in the mesorectum but also in the lateral lymph nodes. The therapeutic approach for the involved lateral lymph nodes remains controversial.

**Objective:**

We performed a systematic review and meta-analysis to assess the prevalence of metastatic lateral lymph nodes in patients with lateral lymph node dissection (LLND) for rectal cancer, which seems to be a fundamental and necessary criterion to discuss any possible indications for LLND.

**Methods:**

Data sources–study selection–data extraction and synthesis–main outcome and measures. We searched MEDLINE, EMBASE and COCHRANE from November 1, 2018, to November 19, 2018, for studies reporting the presence of metastatic lateral lymph nodes (iliac, obturator and middle sacral nodes) among patients undergoing rectal surgery with LLND. Pooled prevalence values were obtained by random effects models, and the robustness was tested by leave-one-out sensitivity analyses. Heterogeneity was assessed using the Q-test, quantified based on the I2 value and explored by subgroup analyses.

**Results:**

Our final analysis included 31 studies from Asian countries, comprising 7599 patients. The pooled prevalence of metastatic lateral lymph nodes was 17.3% (95% CI: 14.6–20.5). The inter-study variability (heterogeneity) was high (I^2^ = 89%). The pooled prevalence was, however, robust and varied between 16.6% and 17.9% according to leave-one-out sensitivity analysis. The pooled prevalence of metastatic lymph nodes was not significantly different when pooling only studies including patients who received neoadjuvant treatment or those without neoadjuvant treatment (*p* = 0.44). Meta-regression showed that the pooled prevalence was associated with the sample size of studies (*p* < 0.05), as the prevalence decreased when the sample size increased.

**Conclusion:**

The pooled prevalence of metastatic lateral lymph nodes was 17.3% among patients who underwent rectal surgery with LLND in Asian countries. Further studies are necessary to determine whether this finding could impact the therapeutic strategy (total mesorectal excision with LLND *versus* total mesorectal excision with neoadjuvant radiochemotherapy).

**Supplementary Information:**

The online version contains supplementary material available at(10.1007/s00268-021-05956-1)

## Introduction

Rectal cancer metastasizes to the perirectal lymph nodes contained within the mesorectum and along the iliac arteries [1]. Assessment of lymph node involvement is a strong predictor of recurrence-free survival and overall survival in patients with rectal cancer [2]. Currently, total mesorectal excision constitutes the gold standard for removing perirectal lymph nodes [3]. However, the therapeutic strategy regarding lymph nodes, notably those located along the iliac arteries, may include neoadjuvant radiochemotherapy, as performed in Western countries [3], or lateral lymph node dissection (LLND), as performed in Japan when the lower border of the tumour is located under the reflection of the peritoneum and when it has passed the *muscularis propria* [4].

However, to date, only two randomized controlled trials (RCTs) [5, 6] have compared the outcomes of the two therapeutic strategies in terms of survival, looking at local and distant recurrences and complications. Furthermore, the choice of the best strategy, either LLND or radiochemotherapy or the combination of the two treatments, is difficult to identify, as the prevalence of metastatic lateral lymph nodes in patients with rectal cancer is poorly documented. Indeed, before comparing two therapeutic strategies and their outcomes, it is necessary and more relevant to know if it is legitimate to propose them as a treatment; in other words, if they would even correctly target metastatic lateral lymph nodes (LLNs).

Therefore, our objective was to perform a systematic review and meta-analysis reporting on the prevalence of metastatic lateral lymph nodes in patients with rectal cancer with the hypothesis that this meta-analysis will elucidate the best therapeutic strategy in the current absence of reliable assessment of tumour aggressiveness.

## Materials and methods

The present methodology is in accordance with the Preferred Reporting Items for Systematic Reviews and Meta-Analyses (PRISMA) checklist (Table S1).

### Literature search and study selection

A literature search was conducted in MEDLINE, EMBASE and COCHRANE from inception (DATE??) until November 19, 2018. Keyword combinations are reported in Table S2. Additional records were identified by manually searching the reference lists of the included publications. To be included, studies had to be written in English or French and to report the prevalence of metastatic lateral lymph nodes among patients with rectal cancer who underwent surgery with LLND. LLND was defined by the dissection of nodes included in areas such as the common iliac (IC), internal iliac (II), external iliac (EI), obturator (O), middle sacral (MS) and aorta bifurcation (Ao).

We excluded case series, conference abstracts, letters to the editor and secondary analyses of previously published papers.

### Data extraction

Two independent reviewers (NC and JM) independently selected articles for inclusion and extracted the data according to a pre-established data collection form. Discrepancies were resolved by reaching a consensus with the senior authors (NCB and FR). The following data were extracted: first author; publication year; country where the study took place; study period, after and before 2010 due to modifications of the Japanese Guidelines for LLND; study design; number of patients who underwent LLND, prophylactic: dissection and removal of nodes that seem invaded (enlarged) based on pre- and peri-operative examinations, *versus* therapeutic/curative: removal of all nodes in the “lateral lymph area”; sex; number of patients with metastatic lateral lymph nodes; number of patients who underwent pre-operative radio- and/or chemotherapy; type of neoadjuvant treatment in those patients; and oncological stages of included patients.

### Statistical analysis

Models with random effects (DerSimonian and Laird’s approach [7]) were used to combine the prevalence of metastatic lateral lymph nodes across the studies. A logit transformation was applied to prevalence before statistical pooling, and the pooled logit of prevalence was then transformed back. Heterogeneity was assessed by using the I^2^ statistic, and a leave-one-out sensitivity analysis was conducted to check the robustness of the pooled prevalence. Potential sources of heterogeneity were investigated by comparing the pooled prevalence between subgroups of studies. A sensitivity analysis was also conducted excluding stage 4 patients from the denominator and from the numerator of the prevalence because stage 4 patients were assumed to have metastatic lateral lymph nodes (model with random effects). In addition, a meta-regression analysis was conducted to assess the relationship between the sample size of studies and the prevalence with the restricted maximum likelihood method. [8] All analyses were performed with the Meta and Metafor packages for R version 3.3.1 (R Foundation for Statistical Computing, Vienna, Austria).

## Results

### Literature search and study characteristics

**Fig. 1:**
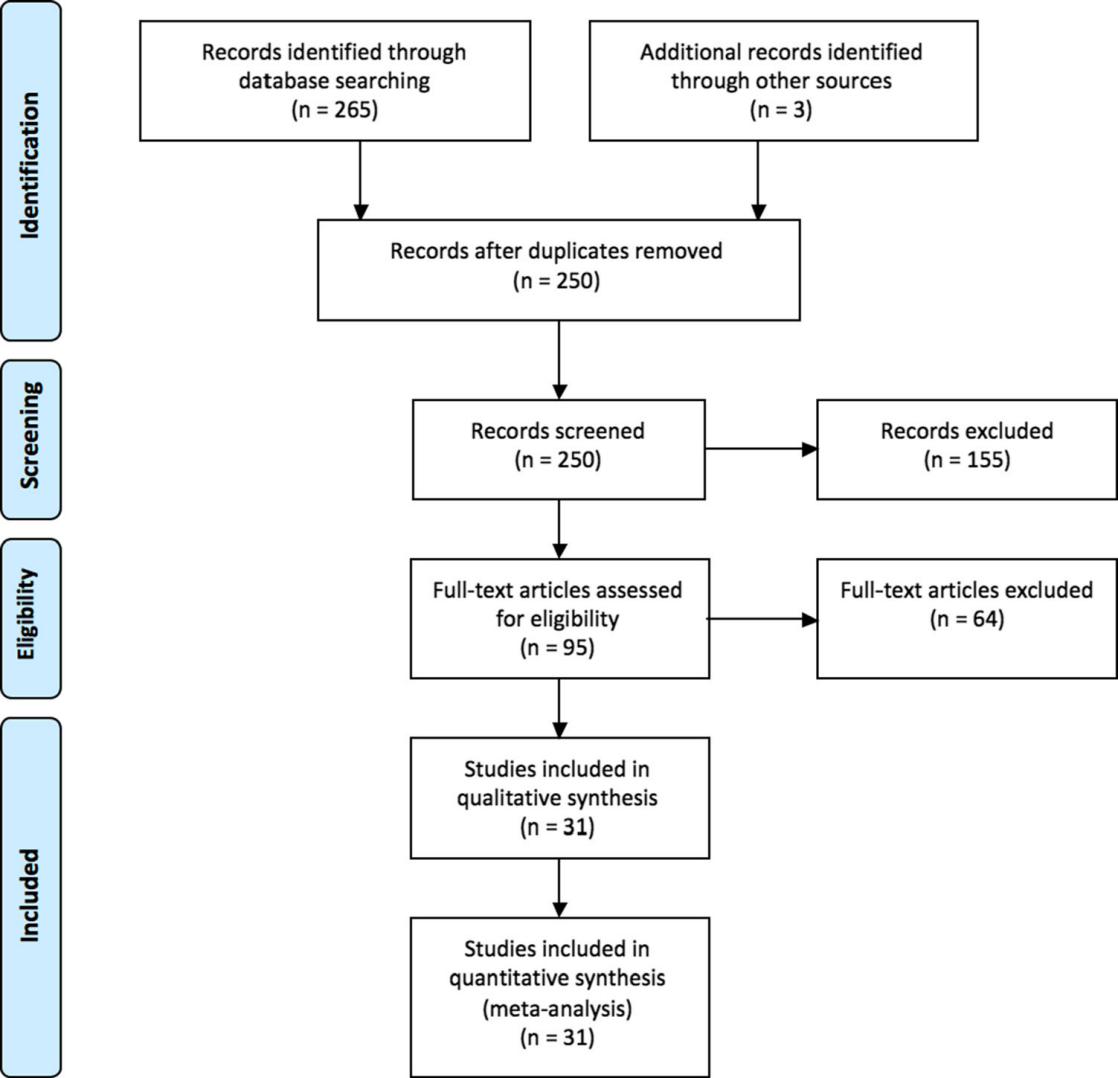
Flow chart showing the selection of publications for quantitative review. Forest plot of the prevalence of metastatic lateral lymph nodes in patients who underwent lateral lymph node dissection. Each horizontal bar summarizes a study. The bars represent 95% confidence intervals. The grey squares represent each of the studies’ weights in the meta-analysis. The diamond in the lower part of the graph depicts the pooled estimate along with 95% confidence intervals. Events = number of patients with metastatic lateral lymph nodes, total = number of patients who underwent lateral lymph node dissection for rectal cancer

Two hundred and sixty-five publications were identified in MEDLINE, EMBASE and COCHRANE. Three publications were identified from other sources. Fifteen duplicates were removed. Of the 250 publications that were identified as eligible, 155 were excluded after title/abstract screening, and 64 were excluded after full-text screening (44 publications did not report the number of patients with metastatic lateral lymph nodes and 20 publications did not distinguish patients with metastatic lateral lymph nodes from those with metastatic lymph nodes from other areas). Ultimately, 31 publications [5, 6, 9–37] were included in the quantitative synthesis, all coming from Japan except one from China (Fig. 1, Table 1).

**Table 1:** Characteristics of included studies

Author	Year	Cohort dates	Design	Mono-/multicentric	Country
Fujita et al	2012	2010–2003	RCT	Multicentric	Japan
Hara et al	2007	1987–1999	Retrospective cohort	Monocentric	Japan
Ishibe et al	2016	2008–2012	Retrospective cohort	Monocentric	Japan
Kanemitsu et al	2017	1975–2009	Retrospective cohort	Multicentric	Japan
Ishida et al	2012	1997–2011	Retrospective cohort	Monocentric	Japan
Kagawa et al	2015	2012–2013	Retrospective cohort	Monocentric	Japan
Masaki et al	2010	2000–2009	RCT	Monocentric	Japan
Matsuoka et al	2007	1997–2005	Retrospective cohort	Monocentric	Japan
Miyake et al	2017	2014–206	Retrospective cohort	Monocentric	Japan
Min et al	2009	1996–2006	Retrospective cohort	Monocentric	Japan
Sato et al	2011	2005–2007	Prospective cohort	Monocentric	Japan
Sato et al	2011	1990–2005	Retrospective cohort	Monocentric	Japan
Shimoyama et al	2003	1981–1994	Retrospective cohort	Monocentric	Japan
Yokoyama et al	2014	2000–2008	Retrospective cohort	Monocentric	Japan
Komori et al	2013	1979–2001	Retrospective cohort	Monocentric	Japan
Masaki et al	2008	2000–2007	Retrospective cohort	Monocentric	Japan
Wu et al	2007	ns	Retrospective cohort	Monocentric	Japan
Ueno et al	2007	1985–2000	Retrospective cohort	Monocentric	Japan
Steup et al	2002	1974–1990	Retrospective cohort	Monocentric	Japan
Mori et al	1998	1975–1996	Retrospective cohort	Monocentric	Japan
Tan et al	2010	1980–2008	Retrospective cohort	Monocentric	Japan
Nagasaki et al	2017	1985–2012	Retrospective cohort	Monocentric	Japan
Yamaoka et al	2017	2013–2015	Retrospective cohort	Monocentric	Japan
Numata et al	2017	2002–2013	Retrospective cohort	Monocentric	Japan
Yamaguchi et al	2016	2010–2014	Retrospective cohort	Monocentric	Japan
Yu et al	2011	2006–2007	Retrospective cohort	Monocentric	China
Kobayashi et al	2009	1991–1998	Retrospective cohort	Multicentric	Japan
Yano et al	2007	1995–2013	Prospective cohort	Monocentric	Japan
Kinugasa et al	2013	1975–2004	Retrospective cohort	Monocentric	Japan
Ueno et al	2005	1985–1999	Retrospective cohort	Monocentric	Japan
Hida et al	1997	1979–1988	Retrospective cohort	Monocentric	Japan

Among the included publications, 28 were retrospective cohort studies [9–35, 37], one was a prospective cohort study [36] and two were RCTs [5, 6]. Thirty studies were performed in Japan [5, 6, 9–33, 35–37] and one in China [34]. Eleven studies only included patients with TNM cancer stages 2 and 3 [5, 9–13, 15, 31, 32, 35, 38]. In 24 studies [5, 6, 9–11, 15, 17–28, 31–35, 37], systematic LLND was performed in all included patients, whereas in six studies [12–14, 16, 29, 30, 36], LLND was performed only in patients with enlarged lateral lymph nodes on pre-operative imaging, which distinguished these patients from others who had confirmation by histopathological results. In one study, the type of LLND was not described [28]. In two studies, patients underwent systematic bilateral dissection [22, 32]; in 27 studies, uni- or bilateral dissection was defined according to clinical and/or imaging findings [6, 12–21, 23, 25, 26, 28–31, 37–39] and in four studies, lateralization of dissection was not documented [24, 34–36]. Nine studies included patients who received neoadjuvant treatment [6, 11, 13–17, 30, 31]. Indications for neoadjuvant treatment were not documented in four studies [11, 15–17] and depended on tumour stage in five studies [13, 14, 16, 30, 31]. The characteristics of the included studies are reported in Tables 2, 3, S3, S4. It is worth noting that the study of Yano et al*.* has been classified above as a study with “curative LLND” because pre-imaging with enlarged nodes was considered the main decision-making criterion for LLND. However, in fact, after the flow chart analysis, dissection was also performed in a systematic way (Table S4). Thus, for statistical analysis, we classified it as a “prophylactic study”.

**Table 2:** Demographics of included studies

Authors	Patients, *n*	Women, *n *(%)	Men, *n *(%)	Age (median)	T1, *n* (%)	T2, *n* (%)	T3, *n* (%)	T4, *n* (%)	Stage 1, *n* (%)	Stage 2, *n* (%)	Stage 3, *n* (%)	Stage 4, *n* (%)
Fujita et al	351	115 (32.8%)	236 (67.2%)	61	n/s	n/s	n/s	n/s	n/s	188 (53.6%)	163 (46.4%)	n/s
Hara et al	177	65 (36.7%)	112 (63.3%)	56.0	n/s	60 (33.9%)	102 (57.6%)	15 (8.5%)	32 (18.1%)	36 (20.3%)	109 (61.6%)	n/s
Ishibe et al	84	31 (36.9%)	53 (63.1%)	62	n/s	n/s	n/s	n/s	8 (9.5%)	25 (29.8%)	39 (46.4%)	12 (14.3%)
Kanemitsu et al	1191	419 (35.2%)	772 (64.9%)	57	26 (2.2%)	348 (29.2%)	731 (61.4%)	86 (7.2%)	244 (20.5%)	351 (29.5%)	596 (50.0%)	n/s
Ishida et al	47	16 (34%)	31 (66%)	62	n/s	n/s	n/s	n/s	11 (23.4%)	13 (27.7%)	23 (48.9%)	n/s
Kagawa et al	50	8 (16%)	42 (84%)	62	n/s	n/s	n/s	n/s	6 (12%)	11 (22.0%)	28 (56.0%)	5 (10.0%)
Masaki et al	55	16 (29.1%)	39 (70.9%)	n/s	n/s	n/s	46 (83.6%)	n/s	n/s	n/s	n/s	n/s
Matsuoka et al	51	16 (31.4%)	35 (68.6%)	63	n/s	n/s	46 (90.2%)	5 (9.8%)	n/s	n/s	n/s	n/s
Miyake et al	25	8 (32%)	17 (68%)	67	2 (8.0%)	10 (40.0%)	8 (32.0%)	1 (4.0%)	10 (40.0%)	3 (12.0%)	3 (12.0%)	5 (20.0%)
Min et al	151	75 (49.7%)	76 (50.3%)	52.6	n/s	n/s	n/s	n/s	n/s	n/s	n/s	n/s
Sato et al	67	24 (35.8%)	43 (64.2%)	63	n/s	n/s	56 (83.6%)	11 (16.4%)	n/s	n/s	n/s	n/s
Sato et al	149	51 (34.2%)	98 (65.8%)	n/s	n/s	13 (8.7%)	125 (83.9%)	11 (7.4%)	n/s	n/s	n/s	n/s
Shimoyama et al	66			n/s	n/s	n/s	n/s	n/s	n/s	n/s	n/s	n/s
Yokoyama et al	131	40 (30.5%)	91 (69.5%)	n/s	n/s	n/s	n/s	n/s	n/s	n/s	n/s	n/s
Komori et al	88			n/s	n/s	n/s	n/s	n/s	n/s	n/s	n/s	n/s
Masaki et al	41	12 (29.3%)	29 (70.7%)	n/s	n/s	n/s	n/s	n/s	n/s	n/s	n/s	n/s
Wu et al	96	50 (52.1%)	46 (47.9%)	65	n/s	n/s	n/s	n/s	n/s	n/s	n/s	n/s
Ueno et al	244	n/s	n/s	n/s	n/s	n/s	n/s	n/s	n/s	n/s	n/s	n/s
Steup et al	605	n/s	n/s	n/s	n/s	n/s	n/s	n/s	n/s	n/s	n/s	n/s
Mori et al	157	62 (39.5%)	95 (60.5%)	n/s	n/s	n/s	n/s	n/s	n/s	n/s	n/s	n/s
Tan et al	1046	340 (32.5%)	706 (67.5%)	58.6	189 (18.1%)	301 (28.8%)	516 (49.3%)	32 (3.1%)	n/s	n/s	n/s	n/s
Nagasaki et al	371	27 (7.3%)	46 (12.4%)	61	n/s	n/s	66 (17.8%)	n/s	n/s	n/s	n/s	n/s
Yamaoka et al	150	35 (23.3%)	115 (76.7%)	61.5	6 (4.0%)	33 (22.0%)	88 (58.7%)	23 (15.3%)	n/s	30 (20.0%)	79 (52.6%)	17 (11.3%)
Numata et al	229	65 (28.4%)	164 (71.6%)	61	n/s	41 (17.9%)	160 (69.9%)	28 (12.2%)	30 (13.1%)	75 (32.8%)	119 (52%)	5 (2.2%)
Yamaguchi et al	173	39 (22.5%)	134 (77.5%)	n/s	n/s	n/s	n/s	n/s	23 (13.3%)	41 (23.7%)	93 (53.4%)	16 (9.2%)
Yu et al	96	42 (43.4%)	54 (56.3%)	60	n/s	n/s	n/s	n/s	n/s	n/s	n/s	n/s
Kobayashi et al	784	277 (35.3%)	507 (64.7%)	n/s	37 (4.7%)	207 (26.4%)	497 (63.4%)	43 (5.5%)	179 (22.8%)	224 (28.6%)	381 (48.6%)	n/s
Yano et al	109	n/s	n/s	n/s	n/s	n/s	n/s	n/s	n/s	n/s	n/s	n/s
Kinugasa et al	994	349 (35.1%)	645 (64.9%)	n/s	142 (14.3%)	260 (26.2%)	658 (66.2%)	384 (38.6%)	n/s	n/s	n/s	n/s
Ueno et al	237	91 (38.4%)	146 (61.6%)	58			224 (94.5%)	13 (5.5%)	n/s	n/s	n/s	n/s
Hida et al	198	n/s	n/s	n/s	8 (4.0%)	38 (19.2%)	128 (64.6%)	24 (12.1%)	n/s	n/s	n/s	n/s

**Table 3:** Characteristics of studies according to the type of LLND surgery and neoadjuvant treatment

Authors	Neoadjuvant treatment	Surgical approach, n	Areas of LLND	Unilateral/Bilateral	Metastatic lateral lymph nodes, *n* (%)	R0 resection, *n* (%)
Fujita et al	No	Open	IC + II + IE + O + MS	Mixed	26 (7.4%)	n/s
Hara et al	No	n/s	II + MS + O ± Ao bifurcation ± IC ± EI	Mixed	32 (18.1%)	n/s
Ishibe et al	No	n/s	IC + II + IE + O	Mixed	16 (19%)	n/s
Kanemitsu et al	Selected patients	n/s	Ao bifurcation + IC + II + IE + 0	Mixed	92 (7.7%)	n/s
Ishida et al	No	n/s	O + II	Mixed	11 (23.4%)	n/s
Kagawa et al	Selected patients	Robotic-assisted	IC + II + O	Mixed	10 (20%)	n/s
Masaki et al	Yes	Open	IC + II + O	Mixed	11 (20%)	50 (90.9%)
Matsuoka et al	Yes	n/s		Mixed	15 (29.4%)	n/s
Miyake et al	Selected patients	n/s	IC + II + IE + O	Bilateral	4 (16%)	n/s
Min et al	Selected patients	n/s	Ao + I + O	Mixed	36 (23.8%)	133 (88.1%)
Sato et al	Yes	n/s	II + IE + O	Mixed	6 (9%)	n/s
Sato et al	No	n/s		Mixed	64 (43%)	n/s
Shimoyama et al	No	n/s	Ao + II + IE + IC	Mixed	20 (30.3%)	n/s
Yokoyama et al	No	n/s	IC + II + IE + O	Mixed	26 (19.8%)	n/s
Komori et al	No	n/s	IC + II + IE + O	Mixed	14 (15.9%)	n/s
Masaki et al	No	n/s	IC + II + IE + O	Bilateral	10 (24.4%)	n/s
Wu et al	No	n/s		Mixed	14 (14.6%)	n/s
Ueno et al	No	n/s	internal pudendal + IC + II + IE + O + MS	Mixed	41 (16.8%)	n/s
Steup et al	No	n/s	IC + II + O	Mixed	83 (13.7%)	n/s
Mori et al	No	Open	II + EI + O	Mixed	40 (25.5%)	n/s
Tan et al	No	n/s	n/s	Mixed	113 (10.8%)	n/s
Nagasaki et al	Selected patients	n/s	II + EI + O + CI	Mixed	73 (19.7%)	68 (18.3%)
Yamaoka et al	Selected patients	Open: 27 Robot: 115 Laparoscopy: 8	CI, II, O	Mixed	34 (22.7%)	n/s
Numata et al	No	Open: 199 Robot: 28 Laparoscopy: 2	CI, II, O	Bilateral	32 (14%)	229 (100%)
Yamaguchi et al	No	Open: 88, Robot: 85	CI, II, O	Mixed	32 (18.5%)	n/s
Yu et al	No	n/s	CI, II, MS, O, EI	n/s	14 (14.6%)	n/s
Kobayashi et al	n/s	n/s	n/s	n/s	117 (14.9%)	n/s
Yano et al	n/s	n/s	n/s	n/s	21 (53.8%)	103 (94.5%)
Kinugasa et al	No	n/s	n/s	Mixed	59 (13.1)	n/s
Ueno et al	No	n/s	CI, IE, II, middle rectal	Mixed	41 (17.3%)	237 (100%)
Hida et al	No	n/s	CI, O, II, middle rectal	n/s	22 (11.1%)	n/s

Common iliac (IC), internal iliac node (II), external iliac node (EI), obturator node (O), middle sacral node (MS), aorta (Ao)

### Prevalence of metastatic lateral lymph nodes

**Fig. 2:**
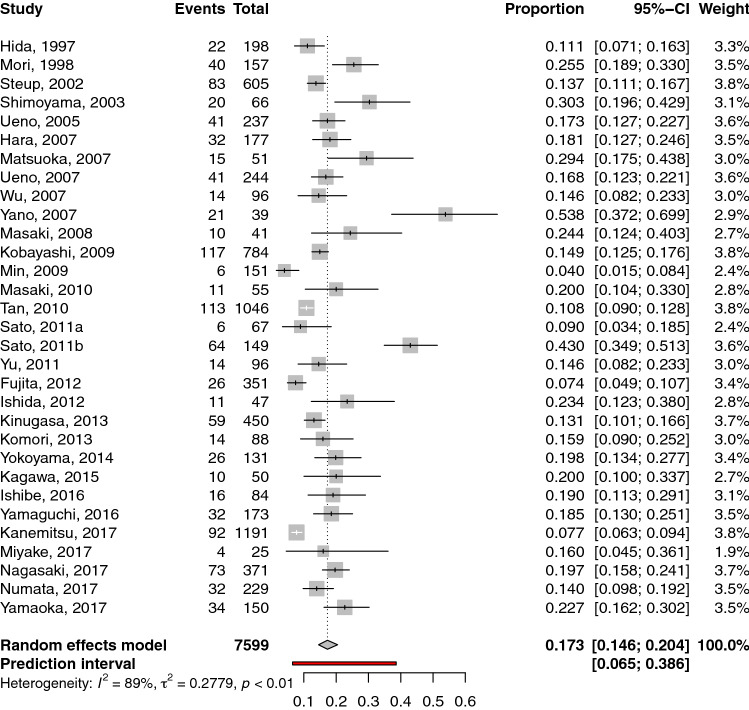
Meta-analysis of the prevalence of metastatic lateral lymph nodes

We obtained a pooled prevalence of metastatic lateral lymph nodes of 17.3% (95% CI: 14.6–20.5) (31 studies, 7599 patients) (Fig. 2). The inter-study variability (heterogeneity) was noteworthy, with I^2^ = 89%. In other words, the variation in study outcome (prevalence) between studies was high. Furthermore, the prediction interval was 6.5 to 38.6%. This means that if a new study is conducted, the assessed prevalence is likely, with a probability of 95%, to fall between 6.5 and 38.6%. However, the pooled prevalence was robust according to leave-one-out sensitivity analysis. The pooled prevalence varied from 16.6% (14.0 to 19.5%), when the study by Yano et al*.* [36] was omitted, to 17.9% (15.1 to 21.1%), when the study by Min et al*.* [16] was omitted (Fig. S1). The heterogeneity, however, remained stable, as the I2 statistic for heterogeneity varied from 85%, when the study by Sato et al*. *[18] was omitted, to 89.1%, when one of the following studies was omitted [6, 10, 13, 15, 21, 23, 25, 26, 32, 34, 35].

To determine whether neoadjuvant treatment could alter the prevalence of metastatic lateral lymph nodes, we performed subgroup analyses by separately pooling studies reporting patients who received neoadjuvant treatment from others (Fig. S2). The difference in pooled prevalence of metastatic lateral lymph nodes between studies with *versus* without neoadjuvant treatment was 16.9% (95% CI: 14.1–20.2), but the inter-study variability (heterogeneity) was high important, with I^2^ = 88%.

Then, we explored whether the heterogeneity could be explained by changes in the therapeutic strategies across the years. The pooled prevalence of metastatic lateral lymph nodes between studies published before and after 2010 was 17.3% (95% CI: 14.6–20.4), but the inter-study variability (heterogeneity) was noteworthy, with I^2^ = 89% (Fig. S3).

Furthermore, we excluded patients with TNM stage IV (in six studies). After exclusion of these patients, the pooled prevalence of metastatic lateral lymph nodes was 15.8% (95% CI 13.1 to 18.9) (Fig. S4).

Then, we separately pooled studies according to the type of LLND performed (systematic *versus* curative). The difference in the pooled prevalence of metastatic lateral lymph nodes between studies with systematic or curative dissection was not statistically significant (*p* = 0.7111) (Fig. S5).

However, the pooled prevalence of metastatic lateral lymph nodes appeared to be associated with the sample size of studies (*p* = 0.005): the prevalence decreased when the sample size increased (Fig. S6). Meta-regression showed that, on average, a study with a sample size that was 10 times larger than that of some other study had an estimated odds ratio for prevalence that was reduced by approximately 50% (Fig. S7).

## Discussion

We performed a systematic review and meta-analysis estimating the prevalence of metastatic lateral lymph nodes in patients with rectal cancer. By pooling 31 studies of Asian origin (30 from Japan and one from China) totalling 7599 patients, we found a prevalence of metastatic lateral lymph nodes of 17.3%, thereby underlining the importance of further examination of the treatment of these node areas to avoid cancer recurrence.

Six studies included patients with TNM stage IV rectal cancer. According to the most recent Japanese guidelines, metastatic disease is not the most important stage to indicate LLND [4]. However, within this stage, some patients may present both primary tumour and resectable distant metastases, thus proceeding to the possibility of undergoing LLND. As a result, to understand the prevalence of metastatic lymph nodes within the most common indications, we excluded patients with TNM stage IV disease by considering them to have metastatic lateral lymph nodes. After exclusion, the pooled prevalence of metastatic lateral lymph nodes remained stable (15.8%).

In the present systematic review and meta-analysis, we were not able to show any improvement in neoadjuvant treatment in terms of lateral lymph node metastasis. However, it should be noted that the power of this subgroup analysis might be insufficient. Additionally, Asian protocols differed from Western protocols, and only a small proportion of patients among studies with neoadjuvant treatment benefited from that modality. Therefore, it is not possible to draw any conclusions about the efficacy of neoadjuvant treatment in terms of the prevalence of metastatic lateral lymph nodes based on our results. Despite this, it is worth noting that neoadjuvant treatments between the studies were quite similar using 5-fluorouracil, oxaliplatin and irinotecan (Table S3).

Some centres performed systematic LLND, whereas others performed LLND in patients with enlarged lateral lymph nodes diagnosed by pre-operative imaging. We pooled these studies separately and found no difference in terms of metastatic lateral lymph nodes. After 2010, as Japanese guidelines changed and recommended limiting LLND for all cT3-T4 tumours with a lower edge below the peritoneal reflection, we reported a subgroup analysis of the pooled prevalence of metastatic LLN according to the publication date. Due to the high heterogeneity, we cannot interpret this result as significant.

Furthermore, we note that meta-regression showed that the pooled prevalence of metastatic lymph nodes was associated with the sample size of studies, as the prevalence of metastatic lymph nodes decreased when the sample size increased. We think that this might be explained by more selective indications for LLND in studies including fewer patients with LLND. Thus, even if the number of studies between curative and systematic LLND intention was not the same, the tendency of the sample size of studies with curative LLND was lower. This indirectly shows that smaller studies were more likely to have a selected population. As a consequence, studies with larger sample sizes (in other words, studies with prophylactic LLND) were more likely to represent the “real” proportion of patients with rectal cancer with LLN metastasis.

Our meta-analysis is the first to examine the prevalence of metastatic lateral lymph nodes, which seems to be an essential prerequisite before considering any treatment. Indeed, recent literature [40–42] directly questions the oncological and functional results of LLND without even first investigating whether it is necessary.

### Advantages of the study

The strengths of our systematic review and meta-analysis are as follows: 1) the inclusion of a large number of studies reporting the prevalence of metastatic lateral lymph nodes based on histopathological analysis and to clarify, for the first time to our knowledge, its subsequent effects in patients operated on for rectal cancer; and 2) the highlighting of the necessity of systematic treatment of lateral lymph nodes due to the high prevalence (17.3%) of LLN metastasis. Indeed, despite the heterogeneity of the studies, which could have impacted the aim of our work, we were able to make some sound calculations due to a specific statistical analysis methodology.

### Limitations of the study

The limitations of the present study are as follows: 1) the high heterogeneity of the calculated pooled prevalence of metastatic lateral lymph nodes, resulting from the quality and heterogeneity of the included publications; and 2) the final analysis included 31 papers, most of which were from Japan and were retrospective studies. Therefore, some patients might have overlapped because these papers were published from some limited leading hospitals. Moreover, it is worth noting that by focussing on patients who have benefited from LLND, a surgical procedure that is most common in Asian countries, selection has been carried out. As a result, it is expected that the selected studies tend to oversample patients with indications for LLND, with these patients being more likely to have advanced disease. Thus, a bias could have been introduced. The percentage of 17.3% of metastatic LLNs could have been overestimated. This element can explain the counter-intuitive (but non-significant) results of a higher prevalence of metastasis in the prophylactic LLND group (17.3%) *versus* the curative group (15.9%).

## Conclusion

In conclusion, this systematic review and meta-analysis of Asian studies indicates that the prevalence of metastatic lateral lymph nodes in patients with low rectal cancer who underwent surgery is 17.3%. This prevalence is of clinical importance, as it may result in cancer recurrence and calls for the systematic treatment of these lymph node areas. Future randomized controlled trials should determine which therapeutic strategy, whether neoadjuvant radiochemotherapy *versus* systematic or imaging-guided lateral lymph node dissection, offers the best improvement in overall and recurrence-free survival.

## Supplementary Information

Supplementary file 1 (DOCX 84 kb)

Supplementary file 2 (DOCX 99 kb)

Supplementary file 3 (DOCX 106 kb)

Supplementary file 4 (DOCX 91 kb)

Supplementary file 5 (DOCX 103 kb)

Supplementary file 6 (DOCX 110 kb)

Supplementary file 7 (DOCX 38 kb)

Supplementary file 8 (DOC 63 kb)

Supplementary file 9 (DOCX 14 kb)

Supplementary file 10 (DOCX 19 kb)

Supplementary file 11 (DOCX 19 kb)

## Abbreviations

LLND: Lateral lymph node dissection
RCT: Randomized controlled trial
