# Supplementary material for: Prevalence of Metastatic Lateral Lymph Nodes in Asian Patients with Lateral Lymph Node Dissection for Rectal Cancer: A Meta-analysis
Source: World J Surg. 2021 Feb 4;45(5):1537–47. doi: 10.1007/s00268-021-05956-1 (PMC8026473; doi:10.1007/s00268-021-05956-1)
Supplement: Supplementary file 1 — (DOCX 84 kb) [file 268_2021_5956_MOESM1_ESM.docx]

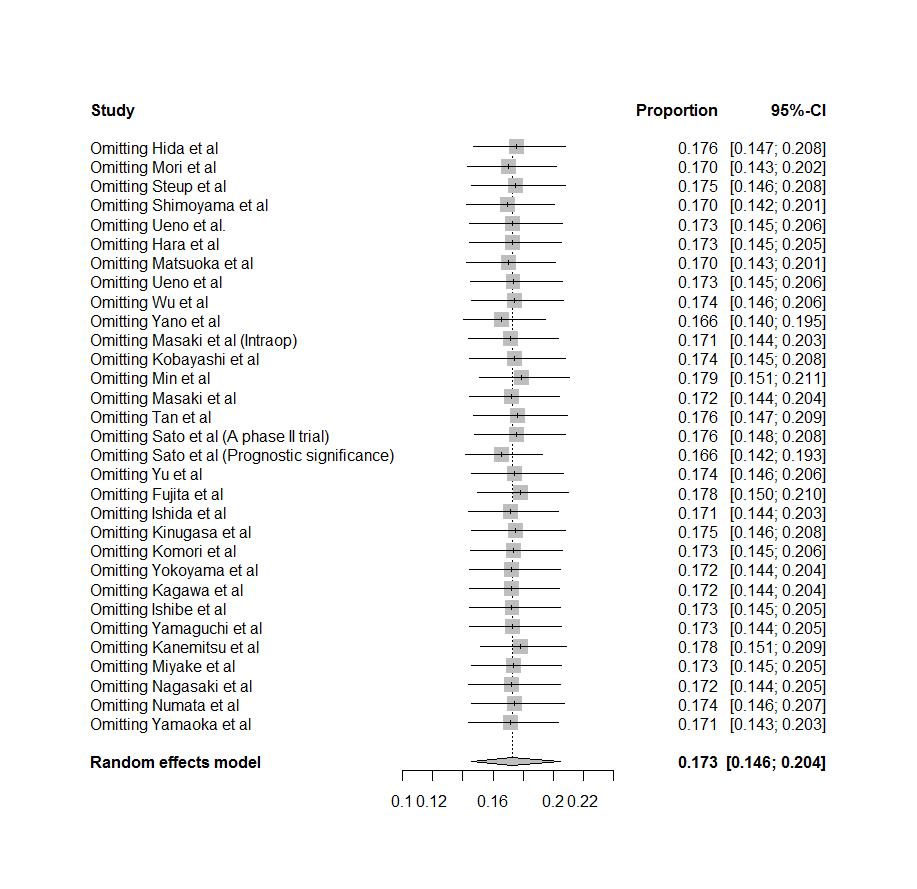


Figure S1. Leave-one-out sensitivity analysis for estimation of the pooled prevalence of metastatic lateral lymph nodes
