# Supplementary material for: Prevalence of Metastatic Lateral Lymph Nodes in Asian Patients with Lateral Lymph Node Dissection for Rectal Cancer: A Meta-analysis
Source: World J Surg. 2021 Feb 4;45(5):1537–47. doi: 10.1007/s00268-021-05956-1 (PMC8026473; doi:10.1007/s00268-021-05956-1)
Supplement: Supplementary file 4 — (DOCX 91 kb) [file 268_2021_5956_MOESM4_ESM.docx]

Figure S4. Sensitivity analysis: subgroup analysis for the pooled prevalence of metastatic lateral lymph nodes among studies including all stages except stage 4


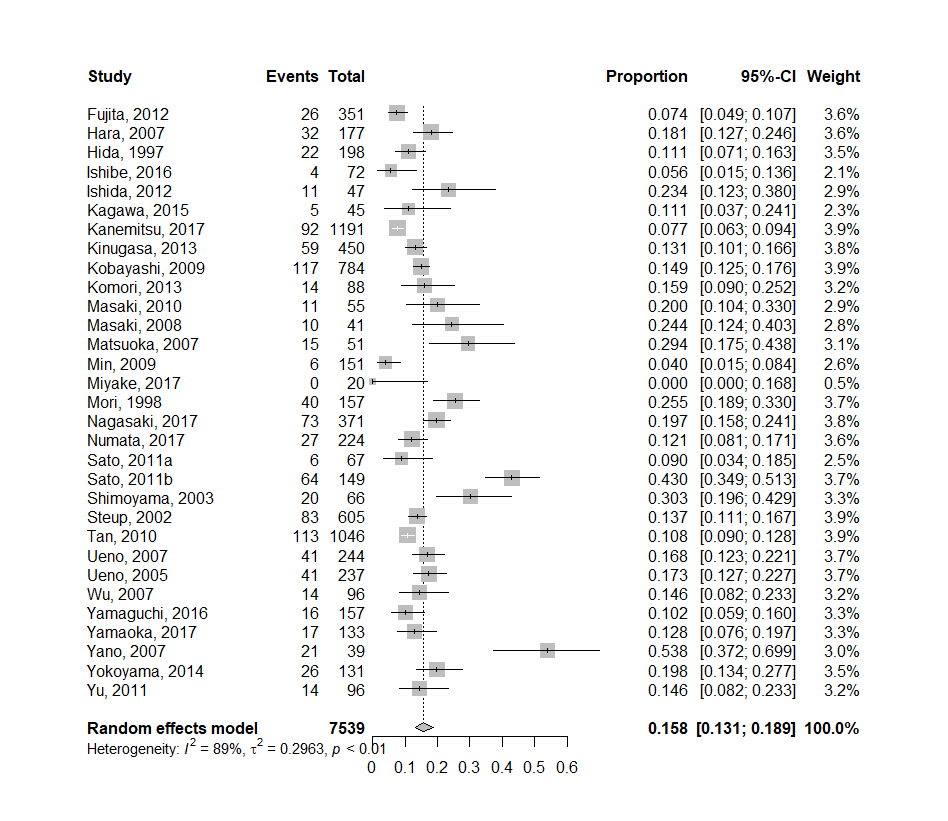


Each horizontal bar summarizes a study. The bars represent 95% confidence intervals. The grey squares represent each of the studies’ weights in the meta-analysis. The diamond in the lower part of the graph depicts the pooled estimate along with 95% confidence intervals. Events = number of patients with metastatic lateral lymph nodes, total = number of patients who underwent lateral lymph node dissection for rectal cancer.
