# Supplementary material for: Prevalence of Metastatic Lateral Lymph Nodes in Asian Patients with Lateral Lymph Node Dissection for Rectal Cancer: A Meta-analysis
Source: World J Surg. 2021 Feb 4;45(5):1537–47. doi: 10.1007/s00268-021-05956-1 (PMC8026473; doi:10.1007/s00268-021-05956-1)
Supplement: Supplementary file 7 — (DOCX 38 kb) [file 268_2021_5956_MOESM7_ESM.docx]

Figure S7. Meta-regression plot


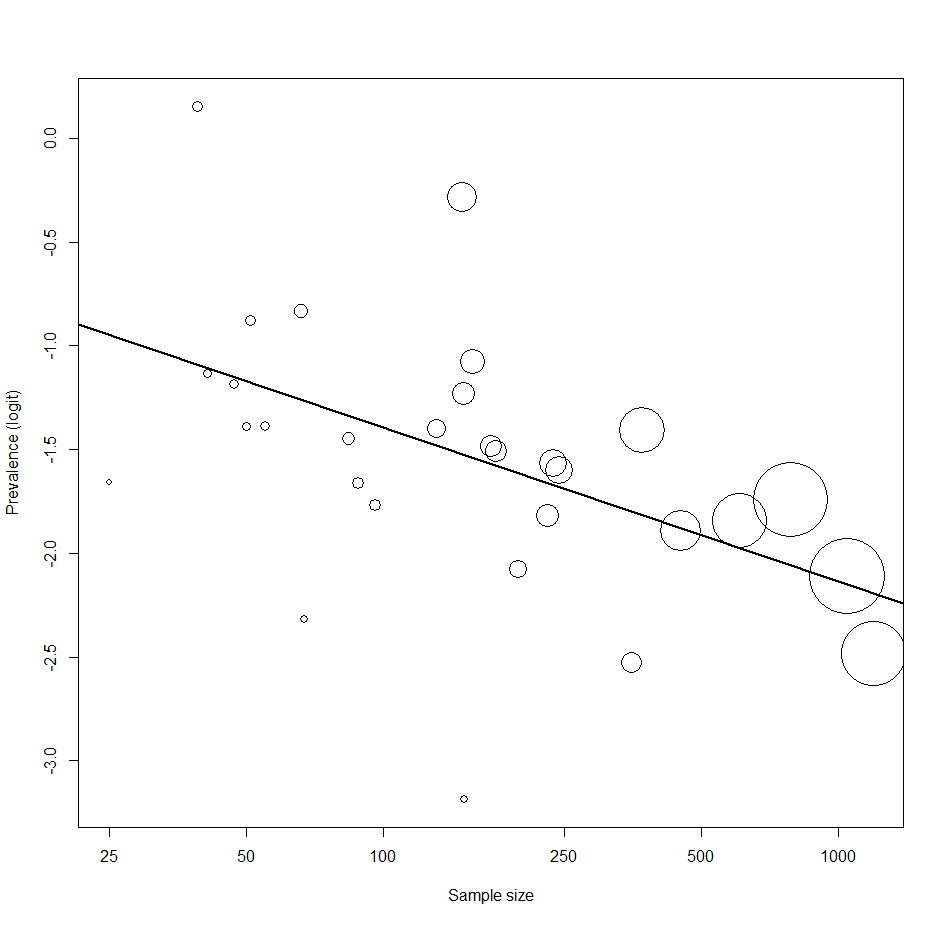


Each circle represents the logit of the prevalence and the sample size of a study. The size of the circle is related to the inverse of the variance of the logit of the prevalence: the larger the circle, the higher is the precision of the estimated prevalence. The black line represent the meta-regression line.
