# Supplementary material for: Prevalence of Metastatic Lateral Lymph Nodes in Asian Patients with Lateral Lymph Node Dissection for Rectal Cancer: A Meta-analysis
Source: World J Surg. 2021 Feb 4;45(5):1537–47. doi: 10.1007/s00268-021-05956-1 (PMC8026473; doi:10.1007/s00268-021-05956-1)
Supplement: Supplementary file 9 — (DOCX 14 kb) [file 268_2021_5956_MOESM9_ESM.docx]

| **Database** | **Search strategy** | **Number of occurrences** |
| --- | --- | --- |
| MEDLINE | ((lateral lymph node dissection[Title/abstract]) OR (lateral lymph-node dissection[Title/abstract]) OR (LLND[Title/abstract]) OR (extended lymphadenectomy[Title/abstract]) OR (extended lymph node dissection[Title/abstract])) AND (rectal neoplasms[MeSH Terms]) | 152 |
| EMBASE | ('lateral lymph node metastasis':ti,ab,kw OR 'extended lymphadenectomy':ti,ab,kw OR 'extended lymph node dissection':ti,ab,kw) AND ('rectum cancer':ti,ab,kw OR 'rectum tumor':ti,ab,kw OR 'mesorectum':ti,ab,kw OR 'mesorectal excision':ti,ab,kw OR 'total mesorectal excision':ti,ab,kw) | 40 |
| COCHRANE | #1 rectal neoplasm (MeSH) and #2 lymph node excision (MeSH) | 73 |

**Table S2. Search strategy**
