# Supplementary material for: Prevalence of Metastatic Lateral Lymph Nodes in Asian Patients with Lateral Lymph Node Dissection for Rectal Cancer: A Meta-analysis
Source: World J Surg. 2021 Feb 4;45(5):1537–47. doi: 10.1007/s00268-021-05956-1 (PMC8026473; doi:10.1007/s00268-021-05956-1)
Supplement: Supplementary file 10 — (DOCX 19 kb) [file 268_2021_5956_MOESM10_ESM.docx]

| Authors | Patients who received chemotherapy neoadjuvant treatment, n (%) | Type of chemotherapy | Patients who received radiotherapy neoadjuvant treatment, n (%) | Type of Radiotherapy |
| --- | --- | --- | --- | --- |
| Kanemitsu *et al.* | 24 (2%) | n/a | 21 (1,8%) | n/a |
| Kagawa *et al.* | 6 (12%) | n/a | 0 | 0 |
| Masaki *et al.* | 0 | 0 | 28 (51%) | Intraoperatively 1 single dose 18-20Gy |
| Matsuoka *et al.* | 0 | 0 | 51 (100%) | Intraoperatively after surgery |
| Miyake *et al.* | 22 (88%) | N=22 whose N(xeloxiri)= 14, N(xelox)=7, N(folfox)=1 | 0 | 0 |
| Min *et al.* | 6 (4%) | 5FU + leucovorin | 6 (4%) | 5040Gy in 25 fractions within 5 weeks |
| Sato *et al.* | 67 (100%) | S-1 and Irinotecan | 67 (100%) | Fractions of 1.8Gy/day for 25 days |
| Nagasaki *et al.* | 30 (8%) | n/a | 30 (8%) | n /a |
| Yamaoka *et al.* | 19 (12,7%) | n/a | 19 (12,7%) | n/a |

**Supplement Table S3: Neoadjuvant treatment in studies including patients who received neoadjuvant radio- and/or chemotherapy**
