# Supplementary material for: Prevalence of Metastatic Lateral Lymph Nodes in Asian Patients with Lateral Lymph Node Dissection for Rectal Cancer: A Meta-analysis
Source: World J Surg. 2021 Feb 4;45(5):1537–47. doi: 10.1007/s00268-021-05956-1 (PMC8026473; doi:10.1007/s00268-021-05956-1)
Supplement: Supplementary file 11 — (DOCX 19 kb) [file 268_2021_5956_MOESM11_ESM.docx]

| Authors | Pre operative imagery  Yes/No | Pre operative Imagery with positive LLN | Prophylactic LLND | Curative LLND |
| --- | --- | --- | --- | --- |
| Fujita *et al.* | Yes (MRI/CT) | No | Yes | No |
| Hara *et al.* | NC | NC | Yes | No |
| Ishibe *et al .* | Yes (MRI) | Yes possible | Yes | No |
| Kanemitsu *et al.* | Yes (MRI, CT, EUS) | Yes possible | Yes | No |
| Ishida *et al.* | Yes | Yes | No | Yes |
| Kagawa *et al.* | Yes (MRI, CT) | Yes | No | Yes |
| Masaki *et al.* | Yes (MRI, CT) | Yes possible | Yes | No |
| Matsuoka *et al.* | Yes (MRI) | Yes | No | Yes |
| Miyake *et al.* | NC | NC | Yes | No |
| Min *et al.* | Yes (MRI) | Yes | No | Yes |
| Sato *et al.* | Yes (MRI) | Yes possible | Yes | No |
| Sato *et al.* | NC | NC | Yes | No |
| Shimoyama *et al.* | NC | NC | Yes | No |
| Yokoyama *et al.* | Yes (CT/MRI) | Yes possible | Yes | No |
| Komori *et al.* | Yes (EUS/MRI) | Yes possible | Yes | No |
| Masaki *et al.* | Yes (CT/MRI) | Yes possible | Yes | No |
| Wu *et al.* | NC | Yes possible | Yes | No |
| Ueno *et al.* | Yes (EUS) | Yes possible | Yes | No |
| Steup *et al.* | NC | NC | Yes | No |
| Mori *et al.* | NC | NC | NC | NC |
| Tan *et al.* | Yes (CT/MRI) | Yes | No | Yes |
| Nagasaki *et al.* | Yes (NC) | Yes | No | Yes |
| Yamaoka *et al.* | Yes (MRI) | Yes possible | Yes | Yes |
| Numata *et al.* | Yes (MRI/CT) | Yes possible | Yes | No |
| Yamaguchi *et al.* | Yes (CT/MRI) | Yes possible | Yes | No |
| Yu *et al.* | NC | Yes possible | Yes | No |
| Kobayashi *et al.* | Yes (US/CT) | Yes possible | Yes | No |
| Yano *et al.* | Yes | Yes possible | Yes | Yes |
| Kinugasa *et al.* | Yes (MRI/CT) | Yes possible | Yes | Yes |
| Ueno *et al.* | NC | Yes possible | Yes | No |
| Hida *et al.* | NC | NC | Yes | No |

**Supplement Table S4 : Type of Lateral Lymph Node Dissestion (LLND) and presence of pre operative imagery**
